# A Dissolving Microneedle Design for Poorly Water-Soluble Drugs for Enhanced Skin Permeation and Transdermal Delivery Fabricated Using 3D Printing

**DOI:** 10.3390/mi17030324

**Published:** 2026-03-05

**Authors:** Sung Giu Jin

**Affiliations:** College of Pharmacy, Dongguk University, Goyang 10326, Republic of Korea; sklover777@dongguk.edu; Tel.: +82-31-961-5229; Fax: +82-31-961-5206

**Keywords:** dissolving microneedles, 3D printing, stereolithography, flurbiprofen, geometric design, transdermal drug delivery

## Abstract

Microneedles (MNs) offer a transformative platform for transdermal drug delivery, though balancing structural precision with mechanical robustness remains challenging. This study utilized SLA 3D printing to fabricate high-resolution MN masters, systematically evaluating printing angles (0° to 60°) and aspect ratios to optimize fidelity. A 45° printing angle was found to significantly enhance tip sharpness and insertion efficiency. These optimized structures served as templates for flurbiprofen (FLU)-loaded dissolving MNs (DMNs) fabricated via a bilayered casting method. We investigated the impact of geometric architectures—conical, pyramidal, and star-type—on functional performance. Mechanical testing using Parafilm^®^ M and ex vivo rat skin revealed that the star-type design, possessing the highest vertex count, exhibited superior strength and a 100% penetration rate by effectively concentrating stress at tip edges. Consequently, star-type DMNs achieved the highest cumulative drug permeation (86.9 ± 9.9% in 12 h), outperforming pyramidal (77.8 ± 9.0%) and conical (64.4 ± 10.2%) designs. These findings underscore geometric design as a critical determinant of clinical efficacy, providing a robust framework for precision manufacturing of task-specific MNs for poorly soluble drugs.

## 1. Introduction

The human skin is a complex, multi-layered organ comprising the stratum corneum (SC), viable epidermis, dermis, and subcutaneous fat. In the context of transdermal drug delivery, the SC acts as the primary rate-limiting barrier, protecting the body from external stimuli and preventing excessive water loss. Due to its highly organized lipid-rich structure, the SC significantly restricts the passive diffusion of most therapeutic agents, particularly high-molecular-weight compounds and highly hydrophilic drugs. Consequently, overcoming this formidable biological barrier is essential for enhancing the systemic absorption and therapeutic efficacy of various pharmaceutical ingredients [1,2,3,4]. Microneedles (MNs) represent a transformative approach to transdermal drug delivery, functioning as a hybrid platform that integrates the advantages of traditional transdermal patches and subcutaneous injection [5]. Compared to conventional administration routes, MNs offer significant clinical benefits, including non-invasiveness, painless application, and the feasibility of self-administration [6]. Furthermore, they are cost-effective and capable of enhancing drug bioavailability by bypassing the hepatic first-pass effect. Generally characterized by micro-scale projections with longitudinal dimensions of 100–2000 μm, MNs enable minimally invasive delivery by breaching the SC. Since these structures primarily target the viable epidermis and circumvent the dense network of nerve fibers residing in the dermis, they facilitate a virtually painless administration process [7,8,9]. As a versatile platform technology, MNs create micron-sized pathways that allow even high-molecular-weight therapeutics to cross the SC [10]. Consequently, extensive research is currently underway to utilize MN systems for the systemic delivery of various active pharmaceutical ingredients. Advancements in material science have facilitated the development of several MN platforms, which are generally grouped into five major types based on their drug delivery mechanism: solid, coated, dissolving, hollow, and hydrogel-forming MNs [4]. Solid MNs facilitate drug delivery by creating transient micropores in the skin before the application of topical formulations [11]. Coated MNs, primarily utilized for vaccine delivery, release drug payloads directly from the needle surface [12]. Dissolving MNs (DMNs) encapsulate drugs within a biocompatible polymer matrix, which subsequently dissolves upon insertion into the body [13]. Hollow MNs allow for the direct infusion of large-volume liquid formulations into the dermis, analogous to conventional injections [14]. Among these, DMNs have gained significant attention due to their excellent safety profile and efficient drug-release mechanisms [15].

In this study, flurbiprofen (FLU) was selected as the model therapeutic agent. FLU is a potent non-steroidal anti-inflammatory drug (NSAID) widely utilized for the treatment of rheumatoid arthritis and related inflammatory conditions [16]. According to the Biopharmaceutics Classification System, FLU is classified as a Class II drug, characterized by high permeability but inherently low aqueous solubility [17]. While effective, its oral administration is frequently associated with significant gastrointestinal side effects, such as gastric irritation and peptic ulcers. To overcome these solubility limitations, various pharmaceutical strategies have been explored, including the development of self-emulsifying systems, solid dispersions, microparticles, and inclusion complexes [18,19,20,21]. In the context of MN-based delivery, enhancing the solubility of hydrophobic drugs is paramount for ensuring adequate drug loading within the micro-protrusions and achieving efficient transdermal flux. Consequently, integrating solubilization techniques into the MN platform is essential not only to bypass the gastrointestinal tract but also to overcome the skin barrier, thereby ensuring improved bioavailability and patient compliance for poorly water-soluble therapeutics.

Traditionally, MNs have been fabricated using microelectromechanical system (MEMS) technology, enabling high-precision manufacturing with sub-micron resolution. However, MEMS-based approaches often involve complex, multi-step processes, high equipment costs, and substantial maintenance overheads [22]. Therefore, developing cost-effective and precise manufacturing techniques is essential to advance MN research. As a disruptive paradigm in manufacturing, additive manufacturing (AM) has surfaced as a viable alternative, enabling the swift and cost-effective production of intricate 3D geometries. A variety of AM modalities, most notably fused deposition modeling (FDM), stereolithography (SLA), and digital light processing (DLP), have been extensively documented for their utility in precision MN engineering [23,24,25]. Although photopolymerization-driven methods such as SLA and DLP are capable of achieving superior structural precision, FDM typically faces resolution constraints due to the physical nature of extruding thermoplastic filaments at elevated temperatures. Although SLA and DLP are widely utilized for their speed and economic efficiency, further optimization is required to achieve the necessary resolution for effective skin penetration [26]. In this study, we fabricated high-resolution MN structures in three geometries—cone, pyramid, and star—using the SLA method to determine whether structural characteristics affect the skin permeation of poorly soluble drugs. To optimize 3D printing parameters, we systematically investigated the effects of printing angle and aspect ratio (AR). Using these 3D-printed structures as molds, we fabricated DMNs containing FLU. In this study, we evaluated the mechanical stability and skin permeability of DMNs as a function of their geometrical configurations. Furthermore, we analyzed the correlation between geometrical structures and the resulting mechanical properties, permeation efficiency, and drug delivery in various MN designs to enhance the skin permeation of poorly soluble drugs.

## 2. Materials and Methods

### 2.1. Materials

A Form 3 SLA 3D printer (Formlabs Inc., Somerville, MA, USA) was employed to construct the MNs with proprietary UV resin. Sylgard^®^ 184 (polydimethylsiloxane; PDMS), used for creating the negative molds, was procured from Dow Corning Co. (Midland, MI, USA). Hanmi Pharm (Suwon, Republic of Korea) provided the FLU used in this study. Furthermore, we utilized Polyvinyl alcohol (PVA) and sulforhodamine B from Sigma-Aldrich (St. Louis, MO, USA), along with Polyvinylpyrrolidone (PVP) and sucrose from Samchun (Pyeongtaek, Republic of Korea), as the primary matrix components. All chemical reagents and solvents were of analytical grade and used as received without further purification.

### 2.2. Design and Fabrication of MNs Using 3D Printing

**Figure 1:**
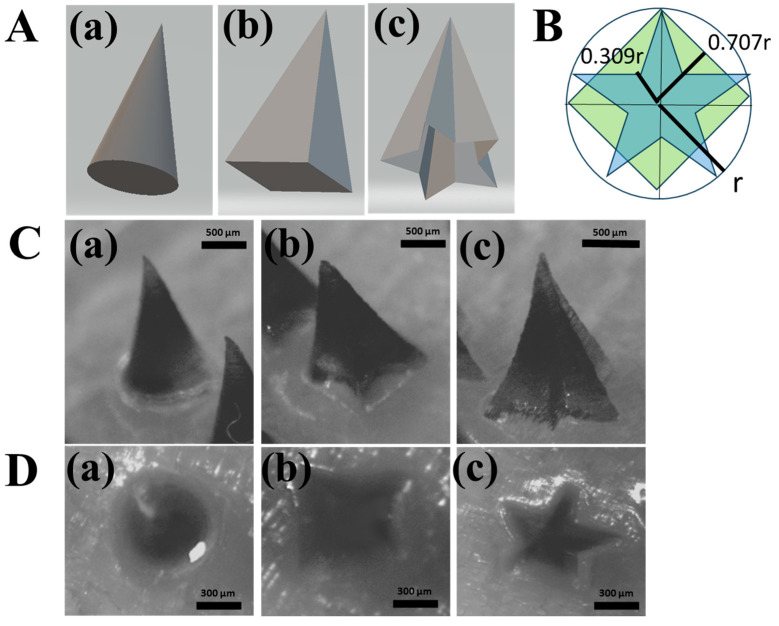
MN structure. (**A**) Design of the MN, (**B**) Minimum distance from the bottom surface and the center of the bottom surface of the MN, (**C**) Representative image, (**D**) Bottom image: (**a**) cone-type; (**b**) pyramid-type; (**c**) star-type.

The geometric architectures of the MNs were designed using computer-aided design (CAD) software (3DS MAX, Autodesk Inc., San Francisco, CA, USA). To investigate the parameters affecting the printing fidelity and structural integrity, the MNs were fabricated with a constant height of 1200 µm, while the base dimensions were varied at 600, 400, and 300 µm. The MNs were arranged in a 6 × 5 array configuration. To optimize the 3D printing resolution and minimize structural deformation, the printing angles were systematically adjusted to 0°, 30°, 45°, and 60°. Three distinct structural models—consisting of conical, pyramidal, and star-type geometries—were established to analyze how morphological differences dictate the mechanical performance of the MNs (Figure 1). Following the 3D printing process, the MNs underwent a standardized post-processing protocol. The fabricated structures were first washed in a dedicated station (Form Wash, Formlabs Inc., Somerville, MA, USA) for 20 min to remove unpolymerized residual resin. Subsequently, the MNs were subjected to UV thermal curing (Form Cure, Formlabs Inc.) at 60 °C for 1 h to achieve complete cross-linking and enhance mechanical strength [26,27].

### 2.3. Fabrication of DMNs

**Figure 2:**
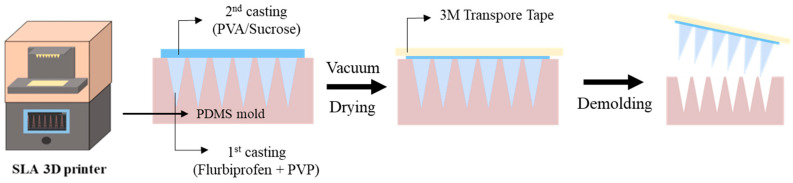
Schematic diagram of the MN manufacturing process.

The DMNs were fabricated using a sequential vacuum-casting process with PDMS molds (Figure 2). For the fabrication of the inverse molds, Sylgard^®^ 184 prepolymer was homogenized with its cross-linker in a 10:1 weight proportion. After pouring the blend onto the SLA-fabricated templates, vacuum evacuation was performed for 30 min to ensure a bubble-free interface at the micro-scale features. Thermal solidification was subsequently achieved by curing the molds in an oven at 85 °C for a duration of 60 min. For the drug-loading layer (the first casting), a solution was prepared by dissolving the FLU model drug in a 5% (*w*/*v*) PVP aqueous solution.

The backing layer solution, comprising 10% (*w*/*w*) PVA and 10% (*w*/*w*) sucrose, was formulated in deionized water for the final casting step. Utilizing a high-performance vacuum pump (MPC601T, Welch, Germany) at −1100 mbar, the DMNs were engineered through a sequential casting process. First, 125 µL of the initial matrix was loaded into the PDMS templates and evacuated for 90 min for precise micro-cavity filling. After a drying stage of 90 min, 300 µL of the secondary (backing) solution was introduced and held under vacuum for 4 h to complete the bilayered structure. Following an overnight solidification phase at room temperature, the DMNs were gently peeled away from the inverse PDMS molds with the aid of 3M medical adhesive tape. To prevent moisture-induced degradation, the fabricated DMNs were maintained in a desiccator for a duration of 24 h before undergoing further morphological and mechanical characterization [13,28].

### 2.4. Morphology and Characteristics of MNs

#### 2.4.1. Morphological Characterization

The structural morphology and surface features of the fabricated DMNs were characterized using a high-performance stereomicroscope (SZ61 TR, Olympus Ltd., Tokyo, Japan). This evaluation was conducted to verify the geometric uniformity, tip sharpness, and overall structural integrity of the MNs across the 6 × 5 array. Digital images were captured to assess whether the replicated DMNs accurately maintained the original dimensions and ARs of the 3D-printed masters [29].

#### 2.4.2. Mechanical Characterization and Insertion Performance

A texture analyzer (MCT-2150; A&D Co., Tokyo, Japan) was employed to assess whether the DMNs possessed sufficient structural integrity and axial strength for successful skin insertion. To simulate the mechanical resistance of human skin, Parafilm^®^ M (Bemis Company, Inc., Neenah, WI, USA) was employed as a validated skin surrogate. The DMNs, secured to a mobile stainless steel probe, were pressed into a pre-arranged 11-layer Parafilm^®^ M assembly (1.3 mm total thickness) immobilized on an aluminum stage. The insertion procedure was conducted under a pre-set force of 32 N with the probe descending at a continuous rate of 20 mm/min. This force was maintained for 1 min to ensure stable penetration. Following the insertion process, the DMNs were withdrawn, and the Parafilm^®^ M layers were carefully unfolded. The penetration efficiency was quantified by counting the number of micropunctures in each layer under a stereomicroscope. This layer-by-layer analysis allowed for the determination of the maximum penetration depth and the distribution of mechanical failure [30,31].

### 2.5. Ex Vivo Skin Penetration Studies

To assess the ex vivo insertion efficiency, excised rat skin was utilized as a biological substrate, which was positioned atop a flat aluminum stage to mimic physiological support. The DMNs were anchored to the texture analyzer’s reciprocating probe and driven into the dermal tissue with a prescribed axial force of 32 N. This compression was executed at a steady displacement velocity of 20 mm/min to ensure reproducible penetration results. This pressure was maintained for 1 min to facilitate consistent insertion of the needles into the tissue. Following the application, the DMNs were removed, and the treated skin site was subjected to histological staining to visualize the penetration sites. Specifically, a 1% (*w*/*v*) methylene blue solution (TCI, Tokyo, Japan) was applied to the skin for 10 min. Methylene blue selectively dyes the micro-channels created by the MNs, distinguishing them from the intact SC. After wiping away the excess dye, the skin surface was observed under a stereomicroscope to analyze the penetration efficiency and the morphology of the resulting micro-conduits [32,33].

### 2.6. Skin Permeation Studies

To determine the transdermal permeation kinetics of FLU across various MN geometries, a vertical Franz diffusion cell apparatus (Hanson Research Corporation, Chatsworth, CA, USA) was employed. The experimental environment was strictly regulated to mimic physiological conditions, with a thermostatic jacket maintaining a constant temperature of 37 ± 0.5 °C. Furthermore, a magnetic stirrer operating at 600 rpm ensured continuous homogenization of the receptor medium throughout the study. With an effective diffusion area of 1 cm^2^, the DMNs were applied to excised rat skin mounted on receptor cells containing 7 mL of PBS (pH 7.4). Sampling was performed at intervals ranging from 0 to 12 h, during which, 300 μL of the receptor fluid was removed and replaced with fresh, isothermal PBS. This rigorous replenishment protocol was strictly followed to ensure that sink conditions were never compromised, allowing for accurate measurement of the drug’s transdermal flux [34,35]. The quantitative analysis of FLU within the receptor fluid was performed using an Agilent 1220 Infinity HPLC system (Agilent Technologies Inc., Santa Clara, CA, USA) equipped with a binary pump (G1312A) and a variable wavelength detector (G1314A). Chromatographic resolution was attained using an Inertsil ODS-3 C18 column (15 cm × 0.46 cm, 5 μm; Tokyo, Japan) maintained at a controlled temperature of 25 °C. The isocratic mobile phase, consisting of phosphoric acid, deionized water, and acetonitrile in a 5:400:600 (*v*/*v*/*v*) ratio, was delivered at a flow rate of 1.5 mL/min. Monitoring was conducted at 254 nm with an injection volume of 10 μL [13,36]. All permeation tests were conducted in triplicate (*n* = 3), and the results are presented as the mean values of steady-state flux and permeability coefficients. The cumulative amount of drug permeated per unit area (µg/cm^2^) was plotted as a function of time (hours). The steady-state flux (Jss) was derived from the slope of the linear portion of the permeation curve according to the following equation:

Jss = (1/A) × (dQ/dt),

where Jss represents the steady-state flux, dQ/dt is the permeation rate at steady state, and A denotes the effective diffusion surface area. Furthermore, the permeability coefficient (P) was determined using the following relationship:

P = Jss/Cd,

where P is the permeability coefficient and Cd refers to the initial drug concentration loaded on the donor side.

### 2.7. Solubility Test

To determine the equilibrium solubility of FLU, an excess amount of the drug (equivalent to 10 mg) was added to microtubes containing 1 mL of distilled water. The mixtures were initially vortexed and subsequently incubated in a water bath at 25 °C with constant stirring at 100 rpm for 5 days to ensure that maximum thermodynamic solubility was reached. Following the equilibration period, each sample was centrifuged at 13,000× *g* for 5 min to remove undissolved particles. The resulting supernatant was carefully collected and filtered through a 0.45 μm nylon membrane filter. The drug concentration in the filtrate was quantified using HPLC, as per the previously described analytical protocol. All solubility measurements were performed in triplicate (*n* = 3) to ensure reproducibility.

## 3. Results and Discussion

### 3.1. 3D Printing Conditions and MN Design Optimization

**Figure 3:**
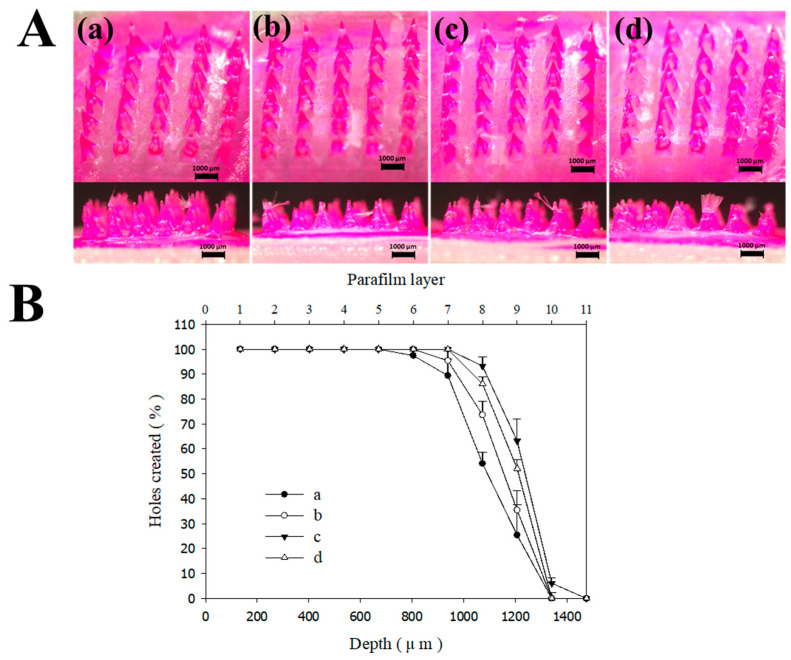
Effect of adjusting the 3D printing angle on the pyramid-type MNs. (**A**) Stereomicroscopic images of the MNs with different printing angles: (**a**) 0°, (**b**) 30°, (**c**) 45°, and (**d**) 60° with both the x and y axes; (**B**) percentage of holes created in each Parafilm^®^ M layer and their corresponding insertion depth.

For effective transdermal drug delivery, MNs must possess the ability to penetrate the SC, which necessitates the fabrication of tips with high structural resolution. Furthermore, MNs must exhibit sufficient mechanical strength to withstand insertion forces, a property intrinsically linked to their geometrical architecture. In this study, we optimized the SLA 3D printing process by adjusting the printing angle to achieve the ideal tip diameter and morphology for drug delivery [23,37]. While SLA 3D printing offers the advantage of rapid prototyping and complex design via CAD software, its primary limitation in MN fabrication has been the difficulty in achieving the sharp tip resolution required for skin penetration [38,39]. During the layer-by-layer additive manufacturing process, the relatively wide base and backing layers of the MNs are typically fabricated with high fidelity to the CAD design. However, as the cross-sectional area decreases toward the apex, the narrowing deposition zone often results in tip blunting or morphological inaccuracies [26,40]. We hypothesized that adjusting the printing angle would alter the deposition orientation, thereby expanding the effective layer area at the tip and improving the structural fidelity. Based on our previous findings [26] where tilting the print axis (x, y, or both) led to sharper tips, we investigated the influence of different printing angles—0°, 30°, 45°, 60°—on the morphology and mechanical integrity of FLU-loaded DMNs using pyramidal-type designs (Figure 3). Attempts were made to fabricate MNs at a 90° orientation, but this angle proved unsuitable for achieving the required tip resolution. Therefore, data for the 90° group were not included in the presented results.

**Figure 4:**
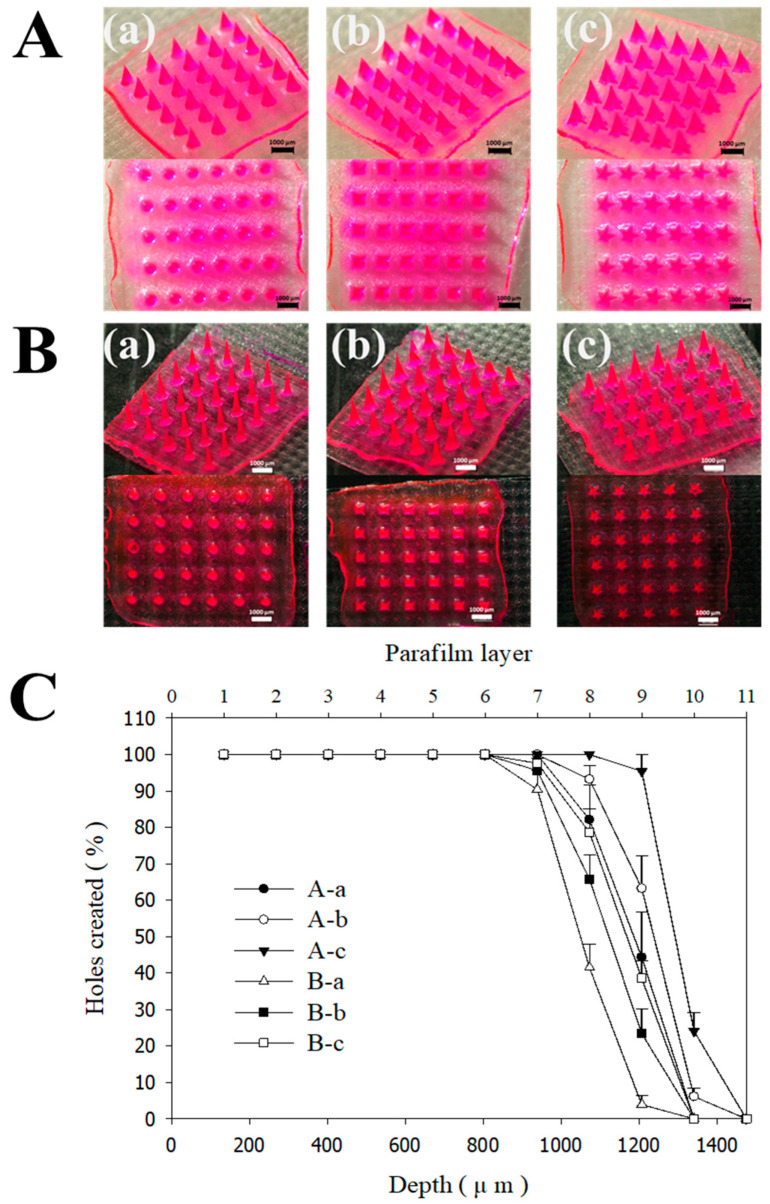
The effect of MN AR on 3D printing according to design. (**A**) Stereomicroscope image with an AR of 2:1; (**B**) Stereomicroscope image with an AR of 3:1; (**C**) Percentage of holes created in each Parafilm^®^ M layer and their corresponding insertion depth. (**a**) cone-type, (**b**) pyramid-type, (**c**) star-type.

To enhance the solubility of the hydrophobic model drug, FLU, we utilized PVP as the primary matrix for the needle tips. PVP is a multifunctional, biocompatible, and hydrophilic polymer widely used as a pharmaceutical excipient due to its excellent solubilizing, binding, and film-forming properties [41,42]. To further reinforce the structural stability of the DMNs, a bilayered configuration was implemented: the drug-loaded tips were composed of PVP. At the same time, the backing layer was fabricated using PVA and sucrose. PVA was selected for its superior mechanical strength and biocompatibility, which provides the necessary robust support for the needles [43,44]. Figure 3A and Figure 3B illustrate the morphological characteristics and insertion efficiency of the MNs, respectively, detailing how the 3D printing angle influences these parameters. The capacity of MNs to penetrate the skin without structural failure or buckling is a critical quality attribute. To evaluate this, we performed axial compression tests using a multi-layered Parafilm^®^ M stack (11 layers) as a skin surrogate, applying a force of 32 N (equivalent to average human thumb pressure) [45]. While all printing angles yielded MNs with generally acceptable macro-morphologies (Figure 3A), significant differences in insertion efficiency were observed (Figure 3B). MNs fabricated at a 0° angle (perpendicular to the build plate) exhibited the lowest penetration capability. In contrast, the insertion efficiency increased at 30° and 45°, with 45° showing the most optimal performance. A slight, non-significant decrease in efficiency was noted at 60°. Additionally, as summarized in Table S1, the tip diameter exhibited a progressive decrease as the printing angle was increased from 0° to 45°. This reduction in tip dimensions directly corroborates the findings of the Parafilm^®^ M insertion assays, confirming that a sharper tip geometry—achieved through optimized printing angles—enhances the mechanical penetration performance of the MNs. These results corroborate previous reports that tilting the print angle optimizes tip sharpness, directly impacting skin penetration performance [26,46,47]. Previous studies have demonstrated that increasing the build angle (e.g., 40–60°) expands the effective layer area of the MN tip, resulting in enhanced sharpness and superior skin penetration. Specifically, Choo et al. identified 60° as the optimal angle for maximizing tip resolution in SLA, while other research has proposed modulating the bevel and tip geometry by adjusting the printing orientation [26]. Furthermore, the angle dependence of fabricated structures was explored by Fitaihi et al., and Jeong et al. successfully controlled the bevel angle through strategic printing direction selection [23,47]. Building upon these precedents, this study applied these principles to the fabrication of PVP polymer matrix structures loaded with poorly soluble drugs. DMNs were fabricated and evaluated using PDMS molds fabricated from 3D-printed masters controlled by the printing angle. These findings reveal that while the gross morphology may not show significant visual divergence, the insertion force—measured via Parafilm^®^ M-based assays—varied significantly with the printing angle. This indicates that the printing orientation is a critical determinant in the manufacture of DMNs, significantly influencing the skin permeability of polymer matrix systems designed for the delivery of poorly soluble compounds. While previous studies utilizing polymer matrices have successfully enhanced drug release, these efforts have primarily focused on water-soluble drugs [48]. For poorly water-soluble drugs, however, the skin permeability is governed not only by the type and concentration of the polymer used but also by the geometric configuration of the delivery system. Although a prior study demonstrated that PVP-based MNs could improve the solubility of FLU [13], the impact of MN architecture and manufacturing design on the resulting transdermal flux remains underexplored. In this study, we emphasize that optimizing the structural geometry is a critical factor in maximizing the clinical potential and penetration efficiency of poorly water-soluble drugs. We investigated the correlation between MN design, AR, and insertion performance (Figure 4). The AR is a pivotal factor influencing both insertion ease and mechanical robustness [26,49,50]. While high-AR MNs (sharper and longer) can facilitate deeper penetration, they are prone to mechanical failure during insertion due to insufficient stiffness. Conversely, low-AR MNs offer higher mechanical strength but may encounter greater resistance during penetration. Given that the optimal AR for DMNs typically ranges from 2:1 to 10:1, we compared three distinct geometries: conical (circular base), pyramidal (square base), and star-type (multi-pointed base) (Table 1 and Figure 1) [26,51].

For this comparison, the printing angle was fixed at 45° (relative to both x and y axes) and the height at 1200 μm. We designed base widths of 600 μm (AR 2:1), 400 μm (AR 3:1), and 300 μm (AR 4:1). Notably, MNs with an AR of 4:1 (300 μm base) failed to produce viable structures, as the needles frequently fractured during the demolding process due to their fragile geometry (Figure S1). Among the successful fabrications, an AR of 2:1 demonstrated superior Parafilm^®^ M insertion efficiency compared to an AR of 3:1. During the multi-layered Parafilm^®^ M insertion test, all designs successfully penetrated up to the 6th layer without structural deformation, demonstrating robust mechanical integrity. Furthermore, the geometric design significantly influenced the results: while conical and pyramidal-type shapes showed similar performance at an AR of 3:1, the pyramidal-type design outperformed the conical-type design at an AR of 2:1. Most importantly, the star-type geometry exhibited the highest insertion capability across all tested ratios. These findings demonstrate that the geometric architecture is a critical determinant of the skin insertion force, which is a key performance indicator for MN-based drug delivery systems [52,53].

### 3.2. Mechanical Hardness and Skin Permeability According to MN Design

**Figure 5:**
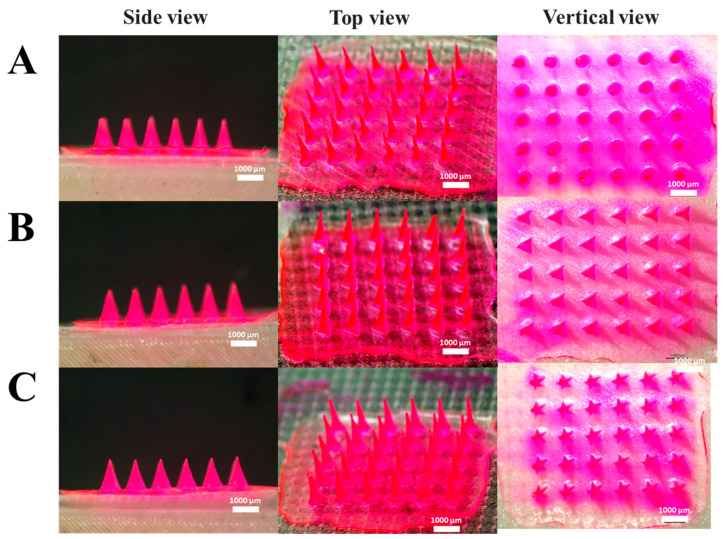
Microscopic analysis of MN structures. Optical microscope images of MN structures for each type, showing both vertical, top, and side views. (**A**) cone-type, (**B**) pyramid-type, (**C**) star-type.

To evaluate the influence of geometric design on functional performance, DMNs were fabricated under optimized 3D printing conditions of a 45° printing angle, 1200 μm height, 600 μm base, and of AR 2:1, with three different geometrical structures: cone, pyramid, and star-type. Figure 5 shows the side, top, and vertical views of DMNs fabricated under the optimal conditions.

**Figure 6:**
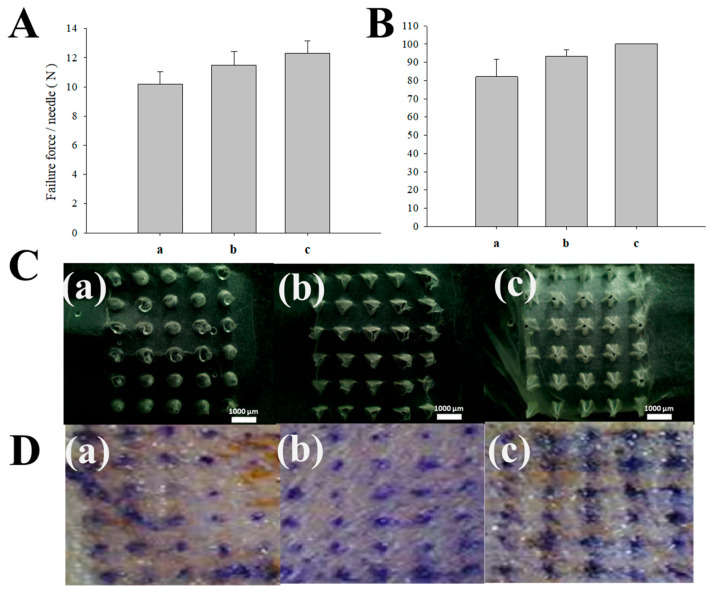
Failure force and insertion capability tests. (**A**) Failure force of MNs; (**B**) Percentage of holes created in 8 layers of Parafilm^®^ M; (**C**) Representative images of Parafilm^®^ M 8th layers after insertion of MNs; (**D**) Skin penetration test of MNs into rat skin ex vivo. (**a**) cone-type, (**b**) pyramid-type, (**c**) star-type.

We systematically compared the mechanical strength and skin insertion efficiency of three distinct geometries (Figure 6). In the mechanical strength evaluation, the star-type DMNs exhibited the highest fracture resistance, followed by the pyramidal and conical designs (Figure 6A). Similar results were observed for the fracture strength of the MNs, as well as their insertion efficiency [54,55]. During the multi-layered Parafilm^®^ M insertion test, all designs successfully penetrated up to the 7th layer without structural deformation, demonstrating robust mechanical integrity. However, distinct performance gaps emerged in the deeper layers (8th to 10th layers). As shown in Figure 6B,C, the star-type design achieved the highest number of successful micropunctures in the deeper layers, confirming its superior penetration capability. These findings were further validated through ex vivo rat skin indentation tests to confirm the disruption of the SC. Following a 1-min application, the skin was stained with methylene blue to visualize the resulting micro-channels (Figure 6D) [56]. Consistent with the Parafilm^®^ M results, the conical design showed a 95% penetration rate, while the pyramidal and star-type designs achieved a 100% penetration rate. These findings demonstrate a positive correlation between the complexity of the polygonal base and the MNs’ functional output, where both mechanical robustness and piercing efficiency improve in proportion to the number of base vertices. This enhancement is likely attributed to the edges of the polygonal cross-section, which effectively reduce the resistance encountered during skin indentation by concentrating stress at the vertices [46,57,58]. Furthermore, as summarized in Table 1, the variations in base area across the different designs suggest that a higher pressure per unit area (stress) is exerted under an equivalent applied force. This concentrated pressure likely facilitates more effective skin breaching, potentially resulting in the observed increase in penetration efficiency for designs with smaller or more optimized base geometries. While Parafilm^®^ M insertion assays and ex vivo skin indentation tests are widely recognized as reliable in vitro surrogate indicators for MN performance, supplementary ex vivo penetration depth measurements would facilitate a more comprehensive understanding of the correlation between needle architecture and skin permeability. Such high-resolution spatial data would further elucidate the mechanistic relationship between structural design and transdermal drug delivery efficiency. Furthermore, additional design parameters beyond the scope of this study—such as the inter-needle spacing and the MN arrangement pattern—may also influence skin permeability. These spatial configurations are known to affect the mechanical stress distribution upon insertion and the subsequent diffusion kinetics, representing important avenues for further optimization of MN-based drug delivery systems.

**Figure 7:**
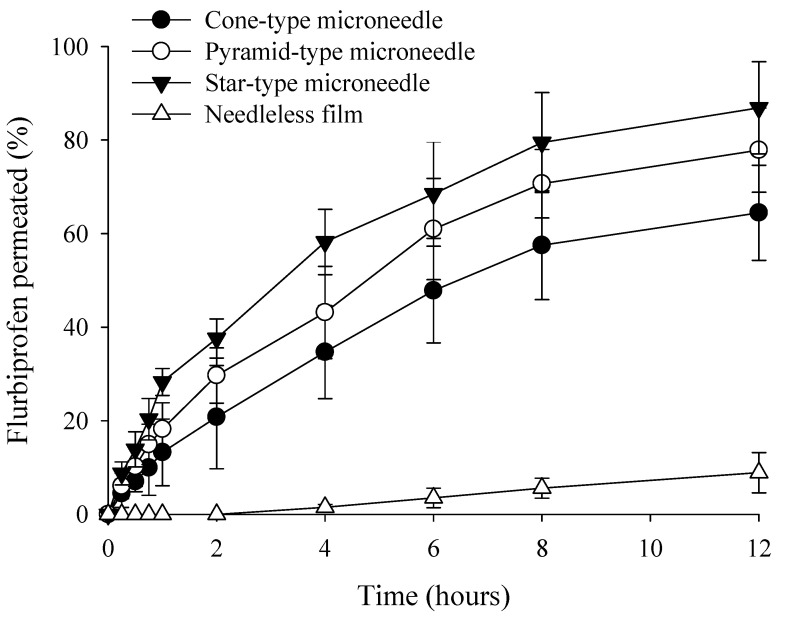
Skin permeability test of FLU transdermal delivery in rats.

Consequently, the star-type design, possessing the highest number of vertices, demonstrated the most effective penetration through the SC, making it the most suitable architecture for transdermal drug delivery. Furthermore, consistent with the results observed for the pyramid-type MNs, the star-type design also exhibited the most favorable insertion force profile at a printing angle of 45° (Figure S2). This suggests that the 45° orientation provides a structural advantage for optimizing penetration performance across different geometric designs. As the body’s most expansive organ, the skin functions as a formidable biological shield, with the SC serving as the principal rate-limiting barrier. To address these transport limitations, we performed 12-h ex vivo permeation assays utilizing a Franz diffusion cell system to evaluate the delivery efficiency across the cutaneous layers (Figure 7). Several studies have previously investigated the delivery of FLU using MN systems. For instance, Chu et al. (2024) developed MNs using genipin-cross-linked gelatin to achieve controlled release rates while mitigating the initial burst effect [59]. Similarly, Mahmood et al. (2021) utilized the hydrophobic nature of FLU to formulate nanoparticles, which were subsequently loaded into MNs [60]. In contrast to these approaches, our study employs a dissolvable PVP polymer matrix specifically engineered to enhance the solubility of poorly soluble FLU. By incorporating the drug within the PVP matrix, the thermodynamic equilibrium solubility of FLU in an aqueous environment was enhanced by more than 14-fold (15.3 ± 1.6 µg/mL compared to its intrinsic solubility of 1.1 ± 0.1 µg/mL). This significant improvement in solubility suggests that the polymer matrix effectively facilitates the solubilization of FLU, potentially maintaining a supersaturated state that drives enhanced transdermal flux.

To further isolate the impact of the physical MN architecture on delivery efficacy, a non-MN FLU-loaded film was utilized as a control. This comparative analysis allowed us to confirm that the observed enhancement in skin permeability is a synergistic result of both the improved drug solubility and the structural advantages of the MN system. In the absence of MN-mediated poration, the skin permeation of FLU remained below 10% over 12 h, confirming that simple passive diffusion is insufficient for effective delivery of this drug. In contrast, all DMN designs significantly enhanced FLU permeability by creating micro-conduits in the skin. The cumulative permeation profiles displayed consistent differences across the geometries throughout the 12-h study. The final permeation percentages for the conical, pyramidal, and star-type designs were 64.4 ± 10.2%, 77.8 ± 9.0%, and 86.9 ± 9.9%, respectively. As summarized in Table 2, the FLU-loaded film (without MNs) exhibited a lag time of 2.23 h. In contrast, all MN designs showed no discernible lag time, suggesting near-instantaneous penetration through the SC. While the permeation profile of the film followed a conventional diffusion-controlled Fickian model, all MN-integrated systems demonstrated significantly accelerated kinetics. The Jss varied according to the structural geometry, following an increasing order from cone-type to pyramid-type, and ultimately star-type designs. This trend aligns with the observed cumulative permeation rates. Notably, the star-type MNs achieved an enhancement ratio approximately 8.5 times higher than that of the control film. Furthermore, as the drug was primarily localized within the needle tips, no significant variations in drug distribution between the tip and the supporting base were observed across the different geometric designs. The star-type DMNs achieved the highest drug delivery efficiency, which is in excellent agreement with our mechanical test results, indicating superior insertion capability [46,61,62].

By increasing the number of vertices in the MN design, we successfully optimized the micro-channel formation and subsequent drug flux. Therefore, the star-type dissolving MN system represents the most promising platform for the enhanced transdermal delivery of FLU. For future clinical translation, comprehensive in vivo safety evaluations are imperative to demonstrate an optimal balance between high permeation efficiency and minimal tissue trauma. Such studies will be crucial in ensuring that the enhanced transdermal delivery achieved by optimized MN designs does not compromise skin integrity or provoke adverse biological responses.

## 4. Conclusions

This study successfully demonstrated that the transdermal permeability of FLU can be significantly modulated by the geometric design of DMNs, which were precisely fabricated using SLA 3D printing. We established that the printing angle and AR in the SLA process are critical parameters that determine the structural fidelity and insertion efficiency of the resulting MNs. Under optimized 3D printing conditions, FLU-loaded DMNs were produced via a robust solvent-casting method. Our results demonstrate that the morphological evolution from a circular base geometry (conical) to a polygonal architecture with a higher vertex count (pyramidal and star-type) yields a marked improvement in both the mechanical stability and the transdermal penetration success of the MNs. This correlation was further corroborated by ex vivo skin permeation studies using rat skin, where the star-type DMN design exhibited superior drug flux compared to other geometries. In conclusion, the geometric architecture of MNs serves as a pivotal factor in governing both mechanical performance and pharmaceutical efficacy. These results suggest that the star-type DMN platform, optimized through additive manufacturing, is a highly promising system for the efficient transdermal delivery of FLU and potentially other poorly soluble drugs.

## Figures and Tables

**Table 1:** Parameters according to the MN structure.

	Shape	Cone-Type	Pyramid-Type	Star-Type
Base	The minimum distance from the center	r	0.707r	0.309r
	Opening Area	πr^2^	2r^2^	0.93r^2^
	Area ratio	1	0.636	0.297
	Vertex number	0	4	10
	Vertex angle	-	90º	36º
	Base diameter (µm)	600	600	600
Tip	Vertex number	1	1	1
	Tip diameter (µm)	90	90	90
Total	Height (µm)	1150	1150	1150

**Table 2:** Permeation parameters of various types of FLU-loaded MNs.

	Steady-State Permeation Flux (Jss) (µg/cm^2^/h)	Lag Time (h)	Permeation Coefficient (10^−5^ cm^2^/h)	Drug Retention (%)	Fickian Fit
Cone-type	6.76	<0.1	N/A *	34.5 ± 7.5	Non-fickian
Pyramid-type	7.83	<0.1	N/A *	24.7 ± 7.5	Non-fickian
Star-type	7.99	<0.1	N/A *	15.2 ± 6.6	Non-fickian
Needless film	0.92	2.23	9.16	89.3 ± 3.5	Fickian

* Negligible lag time due to rapid initial permeation.

## Data Availability

The data presented in this study are available on request from the corresponding author.

## Supplementary Materials

The following supporting information can be downloaded at: https://www.mdpi.com/article/10.3390/mi17030324/s1, Figure S1: The effect of MN aspect ratio on 3D printing according to design. Stereomicroscope image shows MNs with an AR of 3:1: (A) cone-type; (B) pyramid-type; (C) star-type, Figure S2: Percentage of holes created in each Parafilm^®^ M layer and their corresponding insertion depth using the star-type MNs with different printing angles: (a) 0°, (b) 30°, (c) 45°, and (d) 60° relative to the x and y axes. Table S1. Dimensions of the printed microneedles at different 3D printing angles 0°, 30°, 45°, and 60° (n = 6).
