# Supplementary material for: A Dissolving Microneedle Design for Poorly Water-Soluble Drugs for Enhanced Skin Permeation and Transdermal Delivery Fabricated Using 3D Printing
Source: Micromachines (Basel). 2026 Mar 5;17(3):324. doi: 10.3390/mi17030324 (PMC13028163; doi:10.3390/mi17030324)
Supplement: Supplementary file 1 [file micromachines-17-00324-s001.zip › micromachines-4160657-supplementary.pdf]

Effect of dissolving microneedle design containing poorly water-soluble drug for enhanced skin permeation and transdermal delivery in 3D printing fabrication

Sung Giu Jin[1, ‡]

[1]College of Pharmacy, Dongguk University, Goyang, 10326, South Korea

E-mail: sklover777@dongguk.edu

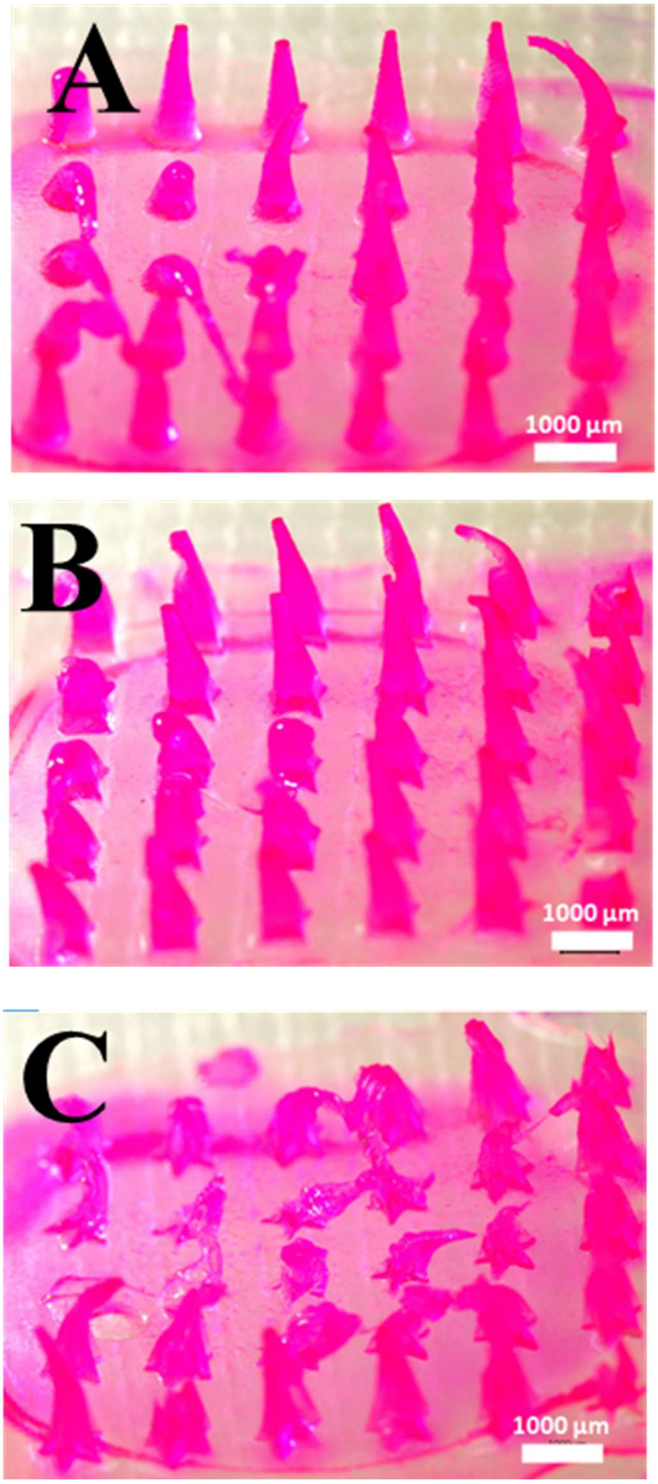

Figure S1. The effect of MN aspect ratio on 3D printing according to design. Stereomicroscope image with an AR of 3:1. (A), cone-type; (B), pyramid-type; (C), star-type.

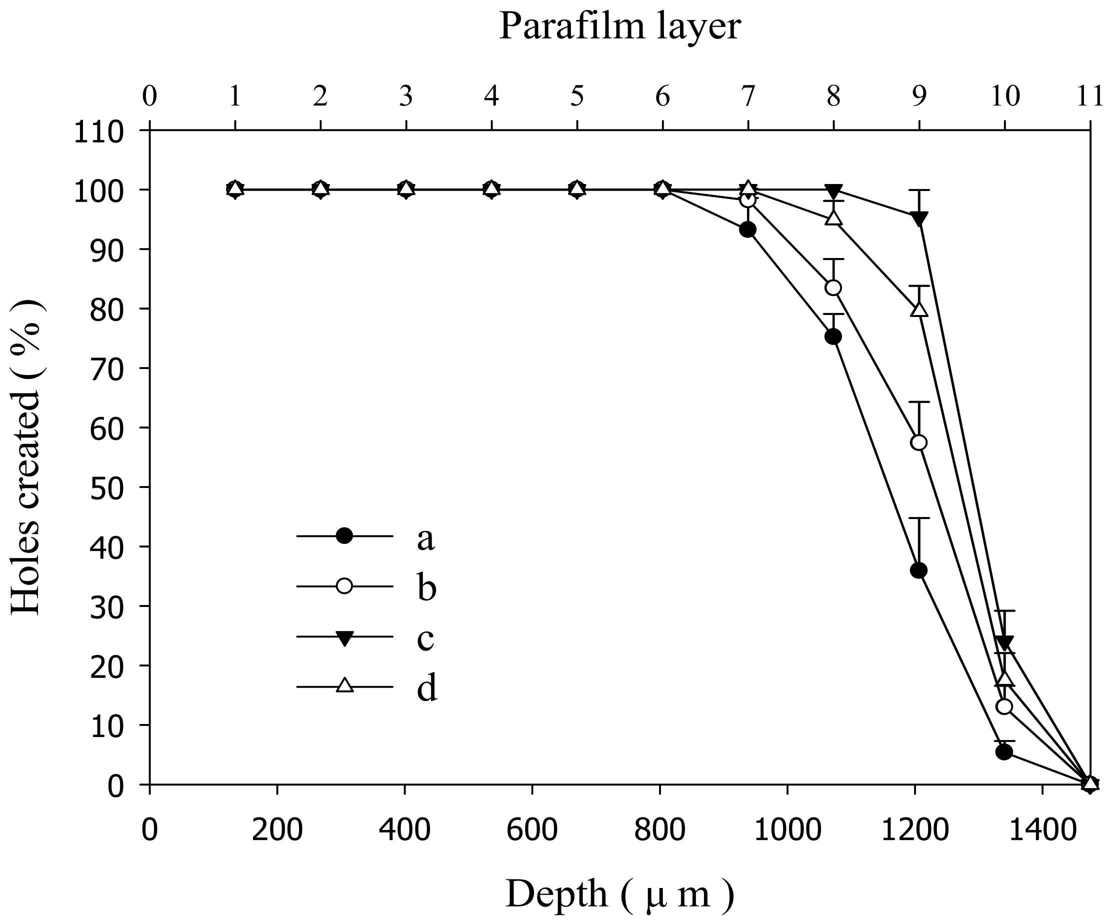

Figure S2. Percentage of holes created in each parafilm® M layer and their corresponding insertion depth of the star-type MN at the different printing angles (a) 0°, (b) 30°, (c) 45°, and (d) 60° to the x and y axes.

Table S1. Dimensions of the printed microneedles at different 3D printing angles 0°, 30°, 45°, and 60° (n = 6).

**Printing Angle**	**0º**	**30º**	**45º**	**60º**
Tip diameter (μm)	165 ± 6	135 ± 7	90 ± 9	95 ± 10
Height (μm)	1,180 ± 10	1,160 ± 10	1,150 ± 10	1,120 ± 10
Base diameter (μm)	600 ± 10	590 ± 20	590 ± 20	590 ± 20
